# Reporting on a Partnership to Co‐Design a Digital Health Intervention With Young People Who Have Experienced Technology‐Assisted Sexual Abuse

**DOI:** 10.1111/hex.70288

**Published:** 2025-05-10

**Authors:** Sandra Bucci, Alice Newton, Pauline Whelan, Kim Cartwright, Prathiba Chitsabsean, Simon Foster, Victoria Green, Amanda Larkin, Rhiannon‐Faye McDonald, Ethel Quayle, Matthias Schwannauer, Victoria Selby, Filippo Varese

**Affiliations:** ^1^ Greater Manchester Mental Health NHS Foundation Trust Manchester UK; ^2^ Division of Psychology and Mental Health, School of Health Sciences, Faculty of Biological, Medical and Health Sciences, Manchester Academic Health Science University of Manchester Manchester UK; ^3^ Division of Informatics, Imaging and Data Science, School of Health Sciences, Faculty of Biology, Medicine and Health, Manchester Academic Health Science The University of Manchester Manchester UK; ^4^ Pennine Care NHS Foundation Trust Ashton‐under‐Lyne UK; ^5^ Marie Collins Foundation London UK; ^6^ School of Health in Social Science University of Edinburgh Edinburgh UK; ^7^ NHS Lothian Edinburgh UK; ^8^ Mersey Care NHS Foundation Trust Prescot UK

**Keywords:** co‐design, digital intervention, digital mental health, technology‐facilitated sexual abuse

## Abstract

**Introduction:**

While Patient and Public Involvement and Engagement (PPIE) is a key element of research best practice across healthcare, the co‐design process for digital health interventions (DHIs) remains under‐reported. This study explores the co‐design process of the i‐Minds DHI, developed for young people exposed to technology‐assisted sexual abuse (TASA), with a focus on advancing PPIE in DHI development. The research team collaborated with a Lived Experience Advisory Group (LEAG) to co‐design the intervention, detailing activities, experiences, benefits, challenges and priorities identified throughout the process.

**Methods:**

The study involved four participatory co‐design workshops and focus groups with LEAG members. Key activities included identifying key features of the app design and content, gathering feedback on prototypes, discussing priorities for the app's function and trial design, and refining the language and content of user‐facing materials. Features were prioritised using the MoSCoW method.

**Results:**

Recruitment of LEAG members, facilitated by the Marie Collins Foundation, emphasised the importance of involving trusted organisations when addressing sensitive topics like TASA, as many young people do not initially recognise themselves as victims. Key findings highlighted the importance of clear communication, structured processes (e.g., Terms of Reference) and financial remuneration for members to promote equity of opportunity. Agile development methods enabled iterative refinement of the app, integrating user feedback in real time. However, time and budget limitations constrained the integration of all desired features, with the MoSCoW method providing transparency in decision‐making.

**Conclusion:**

We offer recommendations for effective PPIE, including prioritising lived experience input early in research, allocating sufficient resources and fostering transparent communication. Despite challenges, such as limited diversity within the LEAG and remote meeting formats, PPIE was considered meaningful by members. This study provides a valuable framework for co‐designing DHIs and improving inclusivity in PPIE efforts, particularly in sensitive research areas like TASA.

**Patient and Public Contribution:**

This study was supported by a LEAG, which undertook the role of partners and was involved in study design, ethical considerations, recruitment, content revision and project oversight. Authors included lived experience members and people with intersecting lived experience and research identities.

## 1 Introduction

Mental health research has moved towards recognising the value of experts by experience, so much so that Patient and Public Involvement and Engagement (PPIE) has become a cornerstone of research best practice [1]. Research funders increasingly require lived experience input into grant applications and details on exactly how people with lived experience will be involved in ensuring research priorities are informed by those who the research is intended to benefit [2]. PPIE allows ‘experts by experience’ to play an active role in the design of the science, research and services that exist to serve them [3]. It is grounded in values of research being carried out ‘by’ or ‘with’ the intended beneficiary of an intervention, rather than ‘to’ or ‘for’ the beneficiary [4, 5], with the aim of redressing the historic power imbalance seen in health research [6], particularly research conducted with vulnerable populations.

Several benefits have been identified when meaningful PPIE is effectively carried out in health research, including improved overall study design, increased participation, clarification of mechanisms of action of an intervention, and early identification of barriers and potential issues that the research team may not recognise or anticipate [7, 8]. Meaningful PPIE is not only of value to the research team; experts by experience have also expressed value in contributing to research. For example, it can be a chance to connect with other like‐minded people, gain experience and new skills, and feel truly heard [9]. One possible barrier to meaningful lived experience involvement is that although several frameworks exist to guide PPIE in health research, such as the INVOLVE guidelines, the MRC framework for developing complex interventions and the person‐based approach, there remains a significant gap in frameworks that specifically address the involvement of young people with lived experience of online sexual abuse in the co‐design of digital health interventions (DHIs). This gap is notable because both the population and the modality require specialised considerations. Young people who have experienced sexual violence may face barriers to engagement related to trust, safety, power imbalances and the risk of re‐traumatisation. Moreover, involving them in DHI design introduces further complexities related to privacy, anonymity and digital literacy. While existing literature offers useful strategies for involving young people in research and for trauma‐informed engagement [10], these approaches are not systematically integrated into digital mental health intervention development. Our study seeks to address this gap by drawing together relevant insights from these domains and applying them in a real‐world trial context.

Various participatory methods for guiding collaboration between researchers and stakeholders have been proposed in the literature [11]. Co‐design has been described as meaningful end‐user engagement in idea generation and design alongside the research team [12, 13]. In a landscape where young people have growing needs in relation to their mental health, co‐design has a valuable part to play in the development of mental health services and interventions by generating innovative ideas that better address end users' needs, resulting in improved success for long‐term outcomes [14, 15]. For instance, the *Horyzons* platform is a DHI co‐designed with young people recovering from first‐episode psychosis and showed sustained improvements in social functioning and reduced relapse risk at 18‐month follow‐up [16, 17]. Similarly, the *SPARX* programme, a gamified co‐designed CBT tool developed with adolescents in New Zealand, was found to be as effective as face‐to‐face therapy for depression, with follow‐up studies indicating continued symptom reduction and improved self‐management over time [17]. These examples highlight how co‐design can produce youth‐centred interventions that support long‐term mental health outcomes by fostering greater engagement and relevance.

Funded by the National Institute for Health and Care Research (NIHR) in the United Kingdom, we co‐designed, developed and tested a DHI in the form of a software application (app) to support young people who have experienced technology‐assisted sexual abuse (TASA). We used a mixed‐methods study design to develop, test and evaluate the DHI. The aim of the i‐Minds DHI is to strengthen young people's capacity to *mentalise*, that is, to understand and reflect on their own and others' thoughts, feelings and intentions, in the context of online interactions. By enhancing this skill, i‐Minds seeks to reduce the risk of further harm by helping young people recognise and navigate online risks more effectively, while also supporting them in managing distress related to TASA experiences. In our non‐randomised clinical feasibility trial, we found that the i‐Minds app was safe, acceptable and associated with promising signals of efficacy on valuable outcomes [9]. Details of the development of the app, our qualitative work and our registered protocol and published outcome paper are reported in detail and published elsewhere [17, 18, 19]. This study aimed to document and critically reflect on the co‐design process of the i‐Minds DHI, developed for young people who have experienced TASA. In line with the United Kingdom's NIHR standards for PPIE [20], the primary objective was to report on the activities, experiences, benefits, challenges and shared priorities identified that emerged through collaboration with the i‐Minds Lived Experience Advisory Group (LEAG), our PPIE group in the i‐Minds project. The study sought to advance understanding of meaningful PPIE in the context of DHI development for vulnerable youth populations.

## 2 Materials and Methods

### 2.1 Project Context

The i‐Minds trial was a non‐randomised, single‐arm feasibility trial (ISRCTN16262847) conducted across NHS services in England and Scotland, and a national e‐therapy provider, to assess the feasibility, acceptability and safety of a 6‐week DHI (i‐Minds) based on mentalisation principles for young people (aged 12–18) with experiences of trauma associated with sexual abuse (TASA). Participants were recruited from CAMHS in Greater Manchester and Edinburgh, and a Sexual Assault Referral Centre in Manchester. Baseline data included demographic, clinical and digital use information, including exposure to online harms. Participants accessed the intervention via their own device or a study handset. Clinical outcomes were measured using brief self‐report tools administered at baseline and post‐intervention. Feasibility outcomes included recruitment and retention rates, intervention engagement and safety monitoring, including the reporting of adverse events.

### 2.2 Design

Following the guidance for reporting by Mbuagbaw et al. [21], this was a meta‐research methodological study. Ethical approval for the i‐Minds programme of work was granted by the National Research Ethics Committee (REC) of West Scotland REC 4 (approval number 22/WS/0083). Qualitative work captured reflections on the co‐design process. Data sources included meeting notes, reflective logs and feedback from LEAG members and research staff. Thematic analysis was used to identify recurring themes across stakeholder experiences. The study followed principles of participatory research, with LEAG contributors involved in shaping study priorities, intervention features and evaluation criteria. This iterative approach allowed for dynamic adjustments based on stakeholder input throughout the development cycle.

### 2.3 Approach

This partnership was an integral part of the i‐Minds project, a mixed‐methods programme funded by the NIHR, aimed at developing and testing a DHI to support young people who have experienced TASA. Adhering to INVOLVE best practice principles and guidance, we established a comprehensive partnership to ensure the interventions were co‐designed with those they intended to benefit. The collaboration included: (i) LEAG: individuals with lived experience of TASA; (ii) Parents and Professionals Advisory Group: comprising NHS and third sector professionals and parents of victims and survivors; and (iii) National Stakeholders Advisory Committee: featuring representatives from voluntary and third sector services, law enforcement, child protective services, social media providers and social media representatives. This inclusive approach ensured that the diverse perspectives and expertise of all stakeholders were incorporated into the development and design of the i‐Minds DHI. In this paper, we primarily report on the partnership with those with lived experience of TASA; that is, the ultimate beneficiaries of the i‐Minds intervention and a vulnerable group that, due to stigma around TASA, presents unique challenges in terms of involvement and partnership with healthcare and research professionals.

Given the sensitive nature of the study topic, comprehensive safeguarding procedures were implemented to support LEAG members throughout their involvement in the project. These procedures were guided by trauma‐informed principles and co‐developed with our partner charity, the Marie Collins Foundation, which specialises in supporting young people affected by TASA. This partnership meant LEAG members were included in decisions about study procedures and ensured they were appropriately supported between meetings. Additionally, appointing a researcher with lived experience to the team was pivotal in successfully recruiting LEAG members and facilitating group sessions. This researcher was a full‐time member of our research group and worked on this project based on their interest, expertise and availability at study initiation. While only one lived experience researcher contributed directly to this project, our wider research group includes several lived experience professionals employed across various projects run by our group. The lived experience researcher was supported by regular supervision from a clinically trained member of staff and mentorship and line management from a senior lived experience research fellow. These supports are part of our broader organisational commitment to creating a safe, inclusive and empowering environment for lived experience researchers and mitigating potential power imbalances within research settings.

The LEAG was supported by this dedicated group facilitator who maintained ongoing communication with members via phone and email between meetings and who was supervised by a senior clinical member of the project team. An optional debrief period was scheduled immediately following each workshop, providing members a safe space to raise concerns or discuss emotional responses to meeting content. In addition, individual follow‐up calls were offered when clarification or additional support was needed. Emotional and psychological support was also available through the Marie Collins Foundation partnership, which provided expertise in responding to distress that may have emerged during the co‐design process. Importantly, feedback or distress expressed by LEAG members was actively incorporated into the ongoing design and delivery of the study. For example, when a member raised concerns about potentially distressing language or content, the research team worked to adapt materials and ensure they were communicated sensitively. These adaptations were documented and reviewed as part of our iterative approach to safeguarding and inclusive participation. This integrated model of support and responsiveness was central to ensuring psychological safety and sustaining ethical engagement with LEAG members. Our approach highlights the importance of flexible, participant‐informed safeguarding strategies in co‐design work involving potentially vulnerable groups.

### 2.4 Sample and Recruitment

LEAG members were recruited through online materials, other research groups across NHS trusts and universities local to the i‐Minds study recruitment sites (Greater Manchester and Edinburgh, the United Kingdom), the Marie Collins Foundation (a specialist organisation that supports survivors of TASA) and our team networks. Our lived experience facilitator developed an information poster to share with interested potential members (see Supporting Information File 1). Some young people wished to be involved but did not want to be a formal member of a group. In these instances, young people were able to contribute at specific points during the co‐design process. In line with NIHR guidelines, all LEAG members were compensated for their participation, including meeting attendance and reading time.

### 2.5 Data Collection

An activity log and reflections about the experiences, benefits and challenges of being involved in the project were kept by the i‐Minds PPIE project lead and PPIE project facilitator. An Agile software development and prioritisation method was used to design and develop the i‐Minds DHI via collaboration with LEAG members and a multidisciplinary team of clinicians, academics, software engineers and designers and underpinned by a team science approach [12]. Each member of the team contributed expertise in areas that were fundamental to the development of the app. The app was technically built by a specialist Digital Health Software Team based at the University of Manchester (https://sites.manchester.ac.uk/digital-health-software-and-platforms/).

**Figure 1:**
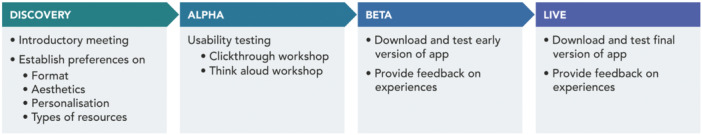
Co‐design phases.

Our approach to the co‐design of the i‐Minds DHI was aligned with the UK Government Digital Service recommendations for User Research (following four co‐design phases, described in Figure 1). The software development of the app followed an iterative Agile software development cycle, with the software team delivering the app iteratively across several Agile Sprint cycles (http://www.agilemanifesto.org), enabling them to respond rapidly to feedback from stakeholders and iteratively refining the app. A cross‐platform app development framework, Ionic (https://ionicframework.com/), was used to develop the app for both Android and iOS mobile operating systems.

The Discovery phase workshop was designed to introduce the technical aspects of the project and establish user preferences regarding the format, aesthetics, personalisation and types of resources required within the i‐Minds app. The Alpha phase workshop aimed to support usability testing of the app prototypes. The Beta phase was designed to support trying out a fully functional version of the app. The Live phase workshop was designed to enable the LEAG to try out the version of the i‐Minds app that would be used in the trial and provide feedback on their experiences.

Following each phase of work, the Digital Health Software team discussed the technical feasibility of identified requirements and identified priorities for the app using the MoSCoW prioritisation method [22]. This method is used in software project management to prioritise deliverables across groups of stakeholders. Using this method, feedback received from stakeholders is categorised as ‘must have’, ‘should have’, ‘could have’ and ‘won't have’. This helps teams focus on the most critical features first while managing expectations about what can be delivered within the constraints of time and budget. Some feedback points were put on hold for implementation due to the complexity of the implementation involved and the time/budget constraints of the project. Software engineers then worked to incorporate the features ranked ‘must have’ and ‘should have’ in the MoSCoW process into the next iteration of the app for presentation at the subsequent phase. The collaboration tool Mural (accessed 18 January 2022, https://www.mural.co/) was used to enable members to share their thoughts on a collaborative whiteboard about the topic discussed.

## 3 Results

### 3.1 Planning, Recruitment and Practicalities

The LEAG included seven members aged 18–30 who all identified as White British females. Between June 2021 and July 2023, we held 13 online (Zoom) meetings. To support each workshop, we provided briefing notes, PowerPoint slides and summaries of previous workshops via email or a pre‐workshop 1:1 meeting with the facilitator, depending on member preferences. Meeting durations ranged from 1 to 2.5 h, with nine meetings specifically focused on the co‐design of the intervention. Input was additionally gathered through virtual participatory workshops, along with various supporting activities and methods designed to empower members to contribute in meaningful and safe ways. For instance, workshops were complemented by other contribution methods, including email, direct input into written documents and individual meetings with research team members.

After recruiting members to the group, we evaluated the most effective way to conduct meetings. Initially, meetings were held online due to Covid‐19 pandemic restrictions. As LEAG members found online meetings convenient for managing their work commitments, reducing travel time and maintaining anonymity (e.g., using the option to keep their cameras off), we continued this format post‐pandemic. One LEAG member chose to keep their camera off during synchronous meetings. However, they remained actively engaged through regular verbal contributions and consistent participation via the chat function. Their well‐being was monitored through optional post‐meeting debrief sessions and individual check‐in calls between meetings, ensuring they had ongoing opportunities to raise concerns and receive support in a format that felt comfortable and accessible to them. Whenever possible, we distributed all meeting materials to group members via email 2 weeks in advance, allowing sufficient time for review. Group meetings consistently followed a structured format: member introductions, establishment or reminder of ground rules (first meeting or subsequent meetings), an overview of the i‐Minds app and study progress, and exploration of a specific topic related to app co‐design or study procedures. Given the group size, we maintained a single group format throughout the meetings instead of using breakout groups. Within the co‐design period, topics of discussion were decided a priori and are shown in Figure 1.

Between meetings, the group facilitator maintained communication with group members, providing updates and requesting additional feedback as needed. The facilitator (A.N.) and the study project manager (A.L./K.C.) attended all meetings. Other research team members, including the project Chief Investigator (S.B.), participated in meetings based on the topic being discussed.

As part of planning for the groups, we held an open discussion with LEAG members about their proposed involvement in the project. To manage members' expectations about involvement, we held an open discussion about the time commitment involved, the importance of confidentiality, ‘code of conduct’ within meetings and establishing ground rules, remuneration for the different types of tasks carried out (based on NIHR INVOLVE guidelines), how notes of meetings will be kept and stored, and the limits of members' involvement. We developed a Terms of Reference document to note these issues and to help manage expectations effectively (see Supporting Information File 2). Through this discussion, we agreed that the LEAG's role would be to contribute to the development of the intervention (including its design, content and functionality), recruitment strategies, participant‐facing documents for the clinical feasibility trial, and the study dissemination strategy.

### 3.2 Co‐Design Workshops and Focus Groups

The LEAG shaped the research in various ways, including: (i) advising the team on the recruitment strategy and troubleshooting recruitment challenges; (ii) developing accessible formats for sharing study results with YP people (and identifying those at high risk and in most need), including the use of video summaries and short, animated information video's explaining more complex topics using inclusive language; (iii) practical advice for encouraging diversity in research participation; (iv) using a tree motif (and other personalisation features) to reflect the user journey and growth through the app; (v) reviewing and advising on the phrasing and language used across the different components of the app and in clinical case examples included in the app content and (vi) providing blog posts to support recruitment/study website. The remainder of this section summarises LEAG's involvement according to the four phases of development we employed.

#### 3.2.1 Phase 1: Discovery Phase

**Figure 2:**
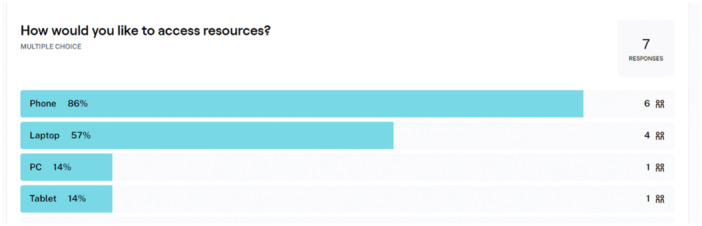
Sample responses about responses to a poll for accessing intervention content (Maze usability task).

The Discovery Phase began with an introductory meeting where we outlined the overall aims of the project. This meeting provided an opportunity for group members to ask questions and decide if they wanted to be involved on an ongoing basis. During this meeting, we established Terms of Reference (Supporting Information File 2) and collectively agreed on the group name (i.e., LEAG) through a collaborative process in which both the research team and LEAG members contributed name suggestions, with the final name selected by majority vote. The next meeting in this phase focused on understanding the needs of young people who have experienced TASA, as expressed by LEAG members. We also gathered feedback via the online platform Maze [23] on various aspects of delivering the intervention, such as the preferred format for accessing the intervention (e.g., phone and laptop; see Figure 2), the app's aesthetic theme, personalisation features and relevant resources to include in the app.

A summary of feedback gathered in this phase is summarised in Table 1, and requirements were prioritised by team members according to the MoSCOW prioritisation method.

**Table 1:** Example MoSCoW features prioritisation gathered in the *Discovery Phase*.

Topic area	Response	MoSCoW prioritisation level	Reason for exclusion
What things do you not want to see in the app?	No signposting to social media	Must have	Yes
	No advertisements in the app	Must have	Yes
What content should we include in the app?	Similar functions to apps that teach breathing exercises	Could have	Resource limitations
	Links to specific resources for different issues related to TASA (e.g., ‘Report Remove’ tool)	Must have	Yes
	Real‐life testimonies from survivors/victims; something people can relate to	Must have	Yes
	A ‘get help’ function and/or ‘quit exit’ button	Must have	Yes
	Features that link social media content to the intervention; it is important to show that not all online spaces are unsafe or bad	Could have	Resource constraints meant a link to social media content could not be moderated
	Information about child services and police (i.e., how to report a crime)	Must have	Yes
	Sources of support that are not diagnosis‐specific	Must have	Yes
	Examples to highlight topic content should display different relationships (e.g., brother, partner and best friends)	Should have	Yes
	Including examples from books and films to illustrate points made (to promote engagement)	Must have	Yes
	Exercises and examples included in the app need clear guidance	Must have	Yes
	Distress and what can ease distress are personal to the individual, so a wide range of exercises is needed	Should have	Yes
	An approved directory of resources	Must have	Yes
How should we present app content?	Pictures/images/videos of real people, to make the app feel more personal	Should have	Yes
	A mixture of exercises (e.g., some passive [listening to a video] and some interactive [breathing exercises])	Must have	Yes
	Interactive games	Could have	Resource limitations did not permit gamification features
	Ensure information is understandable but not patronising	Must have	Yes
What features should we include in the app?	An element of community	Could have	Resource limitations in appointing a moderator
	Diary function to allow people to have a private space where they can express themselves, reflect on how they are feeling and track their mood over time	Should have	Yes
	Ability to interact with other people's stories	Could have	Resource limitations in appointing a moderator; breach of privacy
	Daily reminders with positive messages/inspiring quotes	Should have	Yes
	Live chat function	Could have	Resource limitations in appointing a moderator
Personalisation of the app	The ability to choose colours, wallpapers and so forth to make it feel more personal	Should have	Yes
	Being able to have an avatar with a mixture of choices (e.g., animal and person)	Could have	Resource limitations
	Input daily goals	Could have	Resource limitations
	App content to be filtered by age (as not all information may be understood by all age groups)	Should have	Resource limitations

#### 3.2.2 Phase 2. Alpha Phase

**Figure 3:**
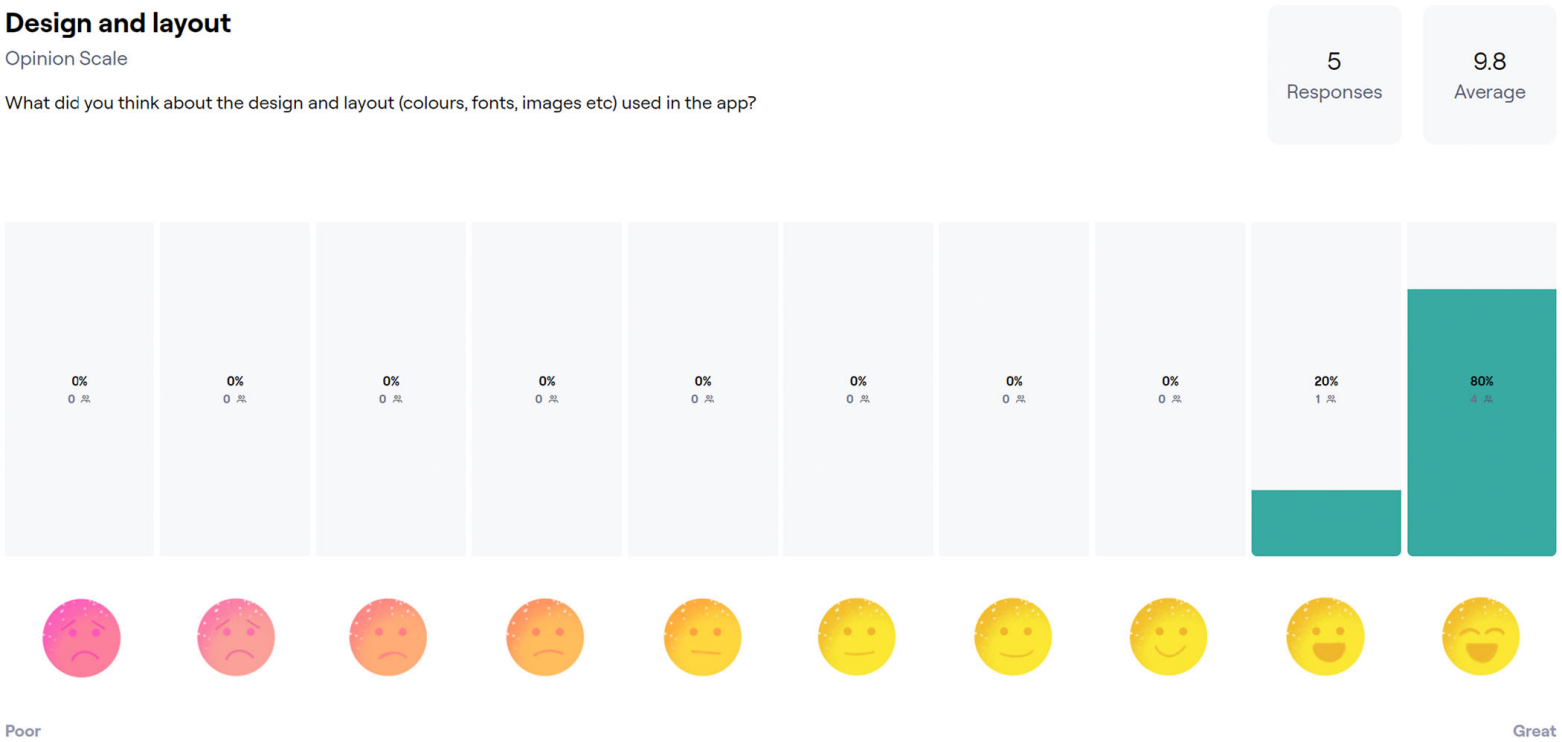
Sample responses to app design and layout using the Maze usability task.

During the Alpha phase, we engaged in various prototyping activities, such as reviewing and refining the layout, design and features of the app. At the first Alpha phase meeting, we held a ‘clickthrough workshop’. This allowed the LEAG to click through the prototype of the app and share their initial thoughts. The second meeting in this phase comprised a ‘think aloud workshop’, which allowed people to work through prototypes of the i‐Minds app on an online platform without the need to download the app onto their own phones. Members were also invited to complete a usability exercise using the online usability testing platform Maze [24] (see Figure 3), accessible with standard computers, tablets and smartphones (Android and iOS supported).

Members used their own devices to access Maze. In this task, group members were invited to complete a series of mini‐tasks asking about people's experience interacting with the prototype. This task allowed us to gather feedback on the acceptability of different aspects of the intervention, for example, the design and layout of the app. Having reviewed the prototypes, members said they liked the design and feel of the app, found it simple and easy to use and found the content engaging. No substantial changes at this stage of development were needed. A summary of the feedback gathered in this phase is shown in Table 2.

**Table 2:** Summary of feedback gathered in the *Alpha Phase*.

Topic area	Response
General feedback so far	The app felt simple to understand and use
	App content was helpful; app content facilitated reflection on feelings without directly telling them to do this
	Some parts of the app were quite wordy, but the content was still easy to understand
	Introduce the term mentalisation earlier in the app, perhaps with an animation or video
	Avoid adding more content to minimise scrolling (potential to reduce engagement)
	Break up the text with imagery
	Add pop‐up check‐ins that offer suggestions on helpful things the young person could do if they are feeling upset
	Members liked the tree concept as it isn't too long or complicated to grow a leaf; it will help to keep people engaged
	Tree concept was not too gendered; I liked its connection to well‐being and growth
	Liked the interactive questions and felt it gave a good break from reading the text
	Include an option to listen to or read content
Thoughts on how to improve engagement with the app	Make the app more personalised instead of one‐way push notifications (e.g., a message asking them to rate progress)
	Include more interactive exercises/text to keep people engaged
	Include a widget or notifications with a quote or idea from the section of the app the young person is currently working on to encourage engagement
	Gamification (e.g., each time a young person opens the app, they receive something, such as points)
	Weekly notifications with a quote or a joke, if they enable notifications
	Ability to like and store content (e.g., exercises the young person liked)
	Make it feel as though the young person is working towards an end goal
Thoughts on the design of the app	Members liked the general layout of the app, colours of the design (it wasn't all blue), illustrations, botanical theme and the tree metaphor
	The app is calming and easy to look at
	Too much black and white on the main screens; include more colours in the colour scheme
Thoughts on the content of the app	Good at ‘breaking down the myths’ in the Introductory pages
	Liked the video content in the app
	Liked the introductory page setting the scene for the app
Thoughts on navigation and flow of the app	Navigation was easy to follow and use
	Positive feedback about the sense of achievement at task completion and the congratulations message at the end
Thoughts on the quizzes and questions in the app	Liked the quizzes and questions and how they prompt reflection
Accessibility of the app	Query about accessibility of font size, can it be changed?

#### 3.2.3 Phase 3. Beta Phase

In the Beta phase, LEAG members had the opportunity to download the prototype app and work through it independently before providing feedback to the group. Members reported experiencing some technical problems with the app (e.g., text being condensed and difficult to read; receiving overly frequent push notifications, page alignment issues) and improvements (e.g., adding a back button, skip option for some material, improve readability in some sections and more variation of genders in the app images), which were fed back to the software development team and subsequently resolved.

#### 3.2.4 Phase 4: Live Phase

**Figure 4:**
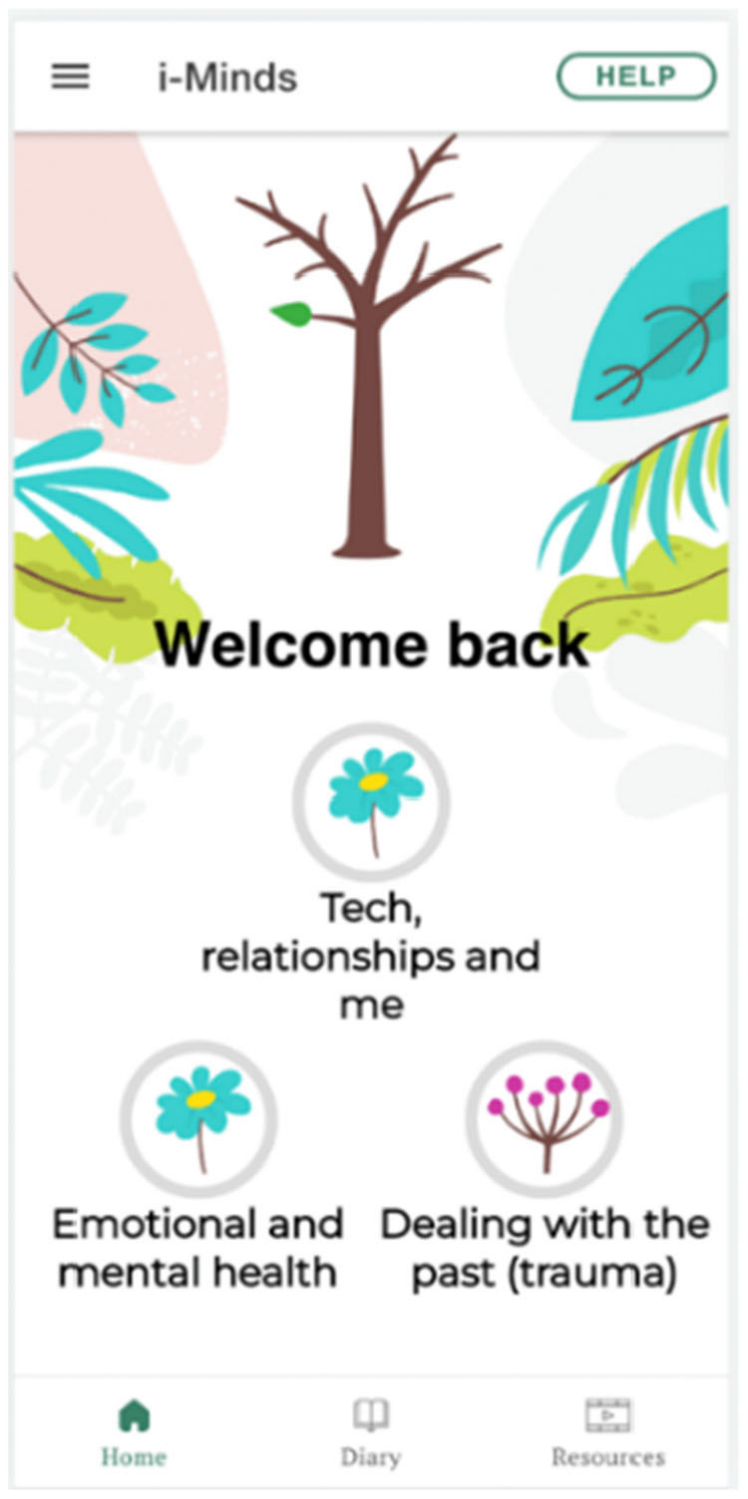
Screenshot of app landing page.

Once the final version of the app was ready for deployment, we invited LEAG members to one final review workshop. At this stage of app development, we emphasised that substantial changes to the app would not be possible, but minor changes could be considered. This was also an opportunity for members to celebrate their work and input to shaping the content and design of the i‐Minds app. A sample image of the i‐Minds app is displayed below (Figure 4), showing how suggestions from the co‐design period came to life in the final live version of the app. The LEAG selected the aesthetics of the app, such as app modules represented by a tree motif. The idea behind the tree metaphor is that it starts out bare and grows leaves as the young person moves through the app and completes modules and activities, symbolising hope and growth.

**Figure 5:**
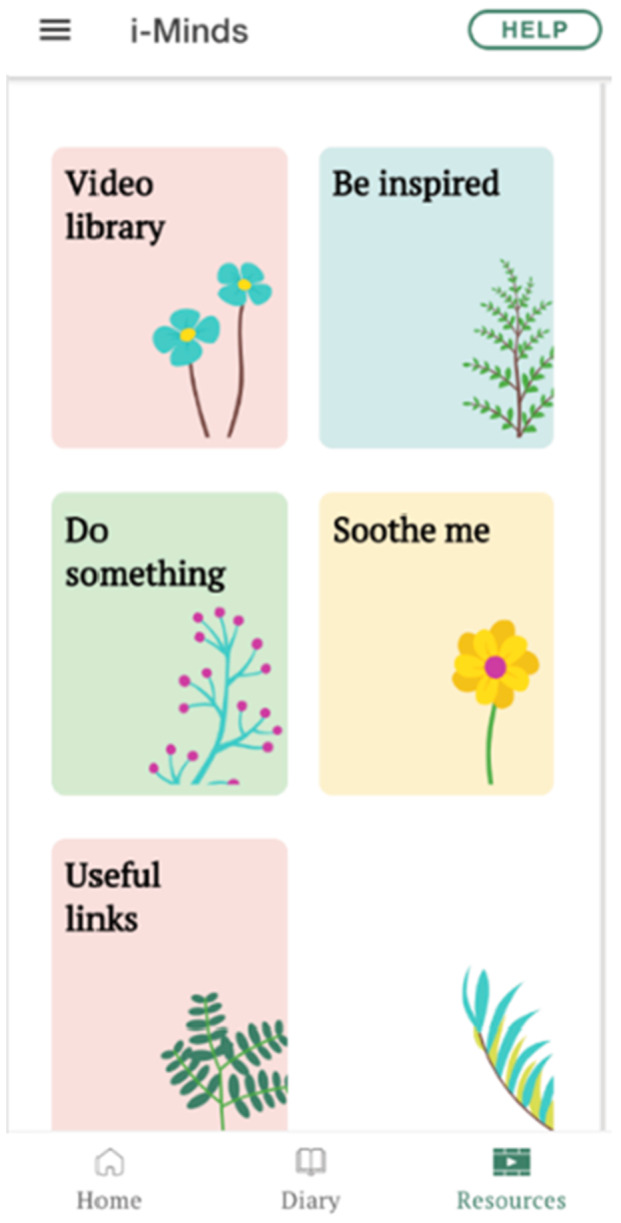
Screenshot of app resources.

The LEAG strongly felt that the app should include a variety of resources (Figure 5) to help users manage potential distress while using the app.

**Figure 6:**
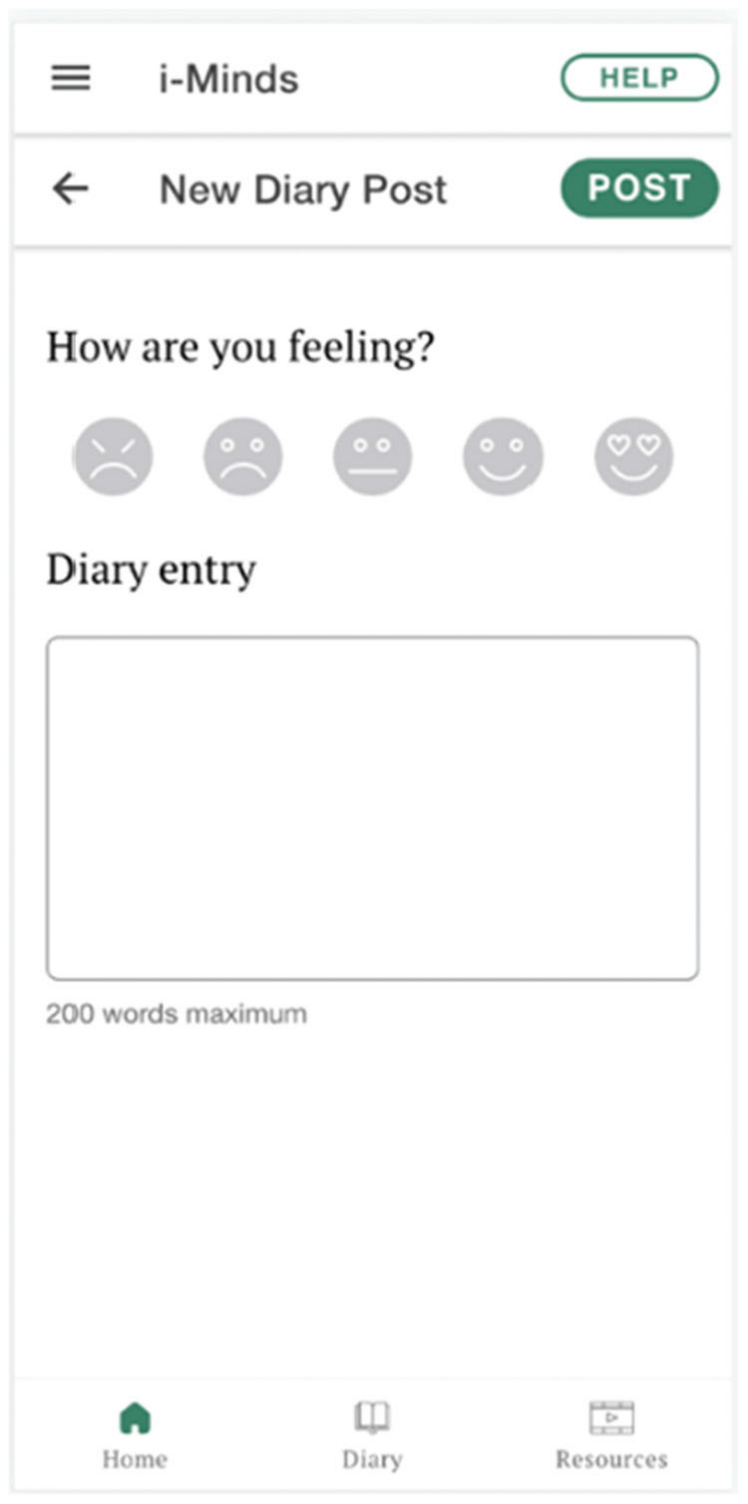
Screenshot of app diary feature.

They recommended incorporating inspiring quotes (e.g., ‘Whatever has happened to you in your past has no power over this present moment because life is now’) and soothing images (e.g., animal pictures) for users to meditate on, as these had provided solace to members during difficult times. The LEAG emphasised the need for an easily accessible help button to ensure users could find support if they felt distressed during the study. They suggested that this help button should be available on every page of the app, not just the home screen. When clicked, it should lead to a list of resources, such as crisis line numbers or contact details of a pre‐identified trusted person. Additionally, LEAG members highlighted the importance of the app's diary function (Figure 6). They reflected on how the ability to diarise their thoughts and feelings as young people had facilitated their own personal journeys of recovery.

### 3.3 LEAG Members and Researcher's Experience of the Benefits and Challenges of Involvement

There was mutual learning from involvement in the i‐Minds programme.

#### 3.3.1 Benefits

We invited LEAG members to reflect on their involvement in the i‐Minds project, both verbally and through written feedback to the group facilitator. They enjoyed participating in all aspects of the project and felt their involvement was meaningful and impactful. They appreciated that their contributions to i‐Minds could help others at risk or directly affected by TASA. This involvement gave them hope for the development of treatments for this vulnerable group. They noted a lack of opportunities to connect with other victims and survivors of TASA and found great value in sharing a space with people who had similar experiences, which provided a sense of connection and normalcy. Knowing that researchers were developing treatments to improve outcomes for young people with TASA gave members a sense of hope.

Research has shown that involvement in research projects can sometimes feel disempowering, tokenistic and isolating [9, 25]. Members described a range of ‘sub‐optimal’ past experiences with research involvement. Some LEAG members had felt ‘used’ and ‘objectified’ in previous research projects. One member mentioned that often, when victims and survivors are asked to be involved in a project, researchers seek specific knowledge or input, usually about the details of their TASA experiences and ‘what was done to us’. Members felt that i‐Minds approached involvement differently. They were not asked to share details of the abuse they had experienced and were made to feel like an integral part of the team throughout the development process, with opportunities to be as involved as they liked. For example, one member highlighted that they were initially asked for written feedback on the language used in one section of the app. However, they wanted the opportunity to provide feedback on the language and content throughout the entire app, and this was accommodated. Members felt that the i‐Minds team took their views seriously, and the impact of their involvement was evident through the MoSCoW prioritisation method. They were regarded as equal partners whose insights were valued as essential to the development process, resulting in a genuinely collaborative approach.

LEAG members valued the consistent communication from the LEAG facilitator. They appreciated receiving regular project updates, especially during times without scheduled meetings. LEAG members felt genuinely appreciated and heard, as they observed the i‐Minds team implementing their feedback and suggestions whenever possible, thanks to the MoSCoW prioritisation method used in the study. They enjoyed seeing their ideas come to life when reviewing the app prototype. Additionally, members liked having various research team members attend meetings, as it allowed them to meet researchers with different roles and expertise.

Members of the research team reflected on the necessity of LEAG's involvement in addressing some of the recruitment challenges encountered and ensuring the app content felt safe, relevant and meaningful. During challenging times, LEAG members reminded the team of the value and importance of the work, providing the motivation and drive to overcome obstacles. Researchers appreciated the positive energy LEAG members brought to the project, which helped maintain staff motivation and kept the focus on the study's broader vision and goals. Researchers also described the privilege of learning from experts by experience about a relatively unspoken and misunderstood topic. They gained insights that extended beyond the i‐Minds project, such as improving recruitment strategies for future related clinical studies.

The Agile software development process enabled iterative versions of the app to be shared with LEAG members, allowing them to see the app evolve and understand their influence on the intervention and study procedures. This iterative approach ensured a continuous cycle of input, refinement and integration of feedback from LEAG members.

#### 3.3.2 Challenges

There were challenges in recruiting LEAG members to the group; this may have been due to it being a group specifically for those who have experienced TASA, a topic that is largely unspoken, often misunderstood and deeply distressing. We also faced challenges in recruiting a diverse group of LEAG members, despite having specialist organisational support to assist with recruitment. After the co‐design phase and the live development of the app were completed, the number and frequency of LEAG meetings decreased and attendance at these meetings also dropped. We sought feedback to understand why this happened. Members indicated that balancing LEAG meetings with their other commitments was challenging. We recognise that experts by experience have various activities and responsibilities outside of PPIE work, often juggling multiple commitments such as work and family life. While these time pressures were identified as key barriers, we also acknowledge that this may not fully explain the reduction in engagement. Feedback from members suggests that involvement in the app design phase was perceived as the most meaningful and impactful aspect of the project—something they prioritised and made time for. The emotionally sensitive nature of the topic, emotional labour involved in participation and possible role ambiguity post the co‐design phase of involvement may also have contributed. Although informal check‐ins were conducted, we recognise the value of more structured feedback mechanisms to better understand and support ongoing involvement. This experience highlights the importance of a flexible and responsive approach to involvement, particularly when working on topics that are emotionally charged or under‐researched. While our Agile software development method allowed us to share iterative versions of the app with LEAG members, it also led to constraints on the features we could implement due to time and resource limitations. Balancing the inclusion of features and functions with milestone deliverables and the funding envelope required making difficult prioritisation decisions at times.

## 4 Discussion

This study detailed the co‐design process of the first DHI for young people exposed to TASA, aiming to advance lived experience involvement in the development of DHIs. It reported on the partnership between the research team and LEAG members, highlighting the activities, experiences, benefits, challenges and key priorities identified in co‐designing the i‐Minds DHI.

Engaging individuals with lived experience of TASA in the co‐design of a digital intervention presented unique ethical and practical challenges. Recruitment was facilitated through collaboration with the Marie Collins Foundation, a specialist organisation with established trust among victims of TASA. This highlights a broader imperative in mental health research: the importance of working in partnership with trusted intermediaries when engaging participants in research on stigmatised and under‐recognised forms of abuse [4]. As noted in previous studies on co‐production with marginalised groups, traditional recruitment methods are often ineffective or inappropriate in contexts where potential participants may not self‐identify with a particular condition or problem or labels and descriptors used in public or generic calls for involvement [4]. In the case of TASA, the complexity is compounded by the digital nature of the abuse—many young people may not perceive their experiences as constituting abuse or may feel silenced by shame, self‐blame or fear of exposure due to the visual and persistent nature of the content involved [26]; that is, young people may not realise that ‘technology‐assisted’ abuse applies to them, so they might not identify with the advert or feel ready to discuss their experiences due to the many silencers around images, videos, chat logs and the increased victim‐blaming associated with TASA. Our experience reinforces existing evidence on the critical role of trust, safety and clarity in enabling meaningful involvement in sensitive research [27]. The presence of a known and trusted intermediary (in this case, the Marie Collins Foundation) allowed potential members to explore involvement without the burden of making an initial disclosure to an unfamiliar research team. Member feedback emphasised that being approached by someone they already knew and trusted was a key factor in their decision to engage. This relational familiarity fostered a sense of safety and autonomy, particularly given the sensitive and often stigmatised nature of TASA. These reflections are consistent with broader literature on sexual abuse disclosure, which highlights that first‐time disclosures are often emotionally complex, shaped by fear of judgement, shame and concerns about not being believed [28]. In addition to this relational foundation, establishing a shared agenda, clear Terms of Reference and appropriate financial recognition were essential structural components that supported equitable and sustained engagement, as highlighted in other PPIE research [29]. These elements, though resource‐intensive, functioned as foundational scaffolding, enabling authentic co‐production [30]. Future work on DHIs targeting trauma‐exposed populations must prioritise both relational and structural conditions as prerequisites for ethical and impactful involvement.

The use of Agile software development methods in this project aligns with a growing body of literature advocating for iterative, participatory approaches in the development of DHIs. Agile methodologies, characterised by short development cycles, regular feedback loops and responsiveness to user input, are increasingly recognised as a means to operationalise co‐production in digital health contexts. In our study, this approach facilitated dynamic collaboration between lived experience advisors, researchers and developers, allowing for visible and iterative changes to the app in response to user feedback. This iterative visibility may have enhanced perceptions of influence and ownership—key factors linked to engagement and satisfaction in co‐designed interventions [4]. However, our experience also illustrates the practical tensions between co‐production ideals and real‐world constraints. Despite the responsiveness afforded by Agile methods, time and budget limitations restricted the extent to which all suggested features could be incorporated. This reflects broader challenges identified in the literature, where participatory approaches risk creating expectations that cannot be met without sufficient structural support [31]. The use of MoSCoW prioritisation provided a transparent framework for managing these tensions, offering a rationale for decision‐making that was shared with group members.

Based on our PPIE work, we make recommendations for conducting meaningful and impactful involvement of lived experience experts in mental health research, drawing on both lived experience and researcher perspectives. To enhance the applicability and theoretical grounding of our recommendations, Table 3 maps each recommendation against established frameworks for meaningful involvement in research, specifically the NIHR UK Standards for Public Involvement [32] and NIHR INVOLVE principles [27], while also highlighting the unique contributions of this study in the context of trauma‐informed mental health research.

1. Prioritise Lived Experience Involvement: Ensure lived experience involvement is prioritised when developing the research idea and during the study set‐up. Ensure their input is considered in planning and decision‐making processes.
2. Allocate Sufficient Resources: Provide adequate time and financial resources to support PPIE across key strategic points of the study. Allocate specific funds for PPIE activities, including compensation for members' time and expenses. Schedule sufficient time for PPIE activities at key strategic points in the study.
3. Plan and Share Timelines: Develop and share comprehensive timelines, meeting notes and relevant documentation with LEAG members to ensure visibility and clear expectations throughout the project and to keep members informed and engaged.
4. Flexible Meeting Schedules: Offer flexible meeting schedules to accommodate members' availability. Use tools like Doodle polls to find convenient meeting times for all members. Offer virtual meeting options to accommodate different schedules and locations.
5. Advance Material Distribution: Send out materials before meetings to ensure members are adequately prepared. Provide clear and concise summaries of complex documents.
6. Transparent Communication: Maintain transparent communication via newsletters, emails and so forth. Clearly explain the PPIE ethos and how it is integrated into the study.
7. Collaborate with Specialist Communities: Partner with specialist communities or organisations, including intersectional community organisations, when recruiting an advisory group to build trust. Engage with communities to understand their needs and concerns.
8. Involve the Entire Research Team: Engage all members of the research team in PPIE meetings at strategic points. Clarify the roles and contributions of each team member in the PPIE process.
9. Move Beyond Extractive Models of Involvement: Approach involvement as a collaborative process, not one that relies on personal disclosures about participants' experiences. Offer lived experience contributors' choice and flexibility in how they participate and recognise their expertise beyond individual experiences. Treat contributors as equal partners, with opportunities to shape both content and process. Allow members to choose how and to what extent they participate. Make transparent how their input informs decisions, to promote trust, respect and shared ownership.
10. Acknowledge PPIE as an Opportunity for Connection: Recognise PPIE as an opportunity for connection and relationship‐building. Create feedback loops to continuously improve the PPIE process based on members' experiences.

**Table 3:** Recommendations for conducting successful and meaningful PPI are mapped where relevant against relevant involvement principles.

Recommendation	Aligned frameworks/principles	Novel contributions from this study
1. Prioritise lived experience involvement	UK Standards for Public Involvement: Inclusive Opportunities NIHR INVOLVE: Respect, Support, Empowerment	Emphasises early involvement in agenda‐setting and decision‐making, particularly important for under‐researched and stigmatised topics like TASA. Meaningful participation must be planned from the outset of a project, with clear consideration of its scope, format and required support. This includes specifying the nature and frequency of involvement, identifying any specialist or safeguarding needs and ensuring that appropriate time and financial resources are allocated in funding applications.
2. Allocate sufficient resources	UK Standards for Public Involvement: Support and Learning NIHR INVOLVE: Reward and Recognition	Stresses the importance of ring‐fenced, trauma‐informed funding and time allocation. Having a well‐planned PPIE budget in i‐Minds allowed us to flexibly expand activities as needed.
3. Plan and share timelines	UK Standards for Public Involvement: Communications	Highlights the need for visibility and clarity to maintain trust and engagement, especially in emotionally demanding contexts. Planning and sharing timelines in advance provided lived experience contributors with a clear roadmap and helps contextualise quieter periods within the overall PPIE process.
4. Flexible meeting schedules	UK Standards for Public Involvement: Working Together NIHR INVOLVE: Flexibility	Emphasises accessibility through practical scheduling to support contributors managing multiple life commitments. Balancing PPIE with work or school commitments can be challenging. In i‐Minds, we accommodated member requests by holding meetings outside standard hours and offering online options to support attendance.
5. Advance material distribution	UK Standards for Public Involvement: Communications	Supports meaningful participation by ensuring contributors are prepared and confident in discussing complex or sensitive content. Where possible, materials were shared two weeks in advance to give i‐Minds LEAG members adequate time to prepare and contribute meaningfully.
6. Transparent communication	NIHR: Communications; Cochrane: Communicate Clearly and Openly	Promotes a clear explanation of the PPIE ethos and how lived experience informs decision‐making throughout the project. Not all feedback can be implemented. We transparently and sensitively communicated what is/is not feasible using the MoSCoW prioritisation method.
7. Collaborate with specialist communities	UK Standards for Public Involvement: Inclusive Opportunities	Highlights the ethical imperative to recruit through trusted intermediaries in contexts involving trauma.
8. Involve the entire research team	UK Standards for Public Involvement: Working Together NIHR INVOLVE: Shared Understanding and Roles	Strengthens team accountability and cohesion by ensuring all researchers are visible participants in the PPIE process. In i‐Minds, various team members, including research staff, the chief investigator, the project manager and designers, attended LEAG meetings, which LEAG members said they valued because of the insight it gave into team roles and contributions.
9. Move beyond extractive models of involvement	NIHR INVOLVE: Respect and Value Lived Experience	Advocates for trauma‐informed, non‐extractive practices that honour diverse ways of contributing beyond personal disclosures.
10. Acknowledge PPIE as an opportunity for connection	UK Standards for Public Involvement: Impact	Recognises the relational and developmental value of PPIE for contributors and researchers, beyond functional project goals. Projects like i‐Minds offer a valuable form of peer support, as many victims and survivors rarely have the opportunity to connect with others who share similar experiences.

This study demonstrates that meaningful PPIE in the design, development and testing of a DHI is both feasible and valuable. The i‐Minds PPIE process was experienced by LEAG members as meaningful, validating and impactful. The four‐step co‐design process, along with the practical insights and recommendations shared, offers a useful framework for future DHI co‐design efforts.

However, there were some limitations. Although in‐person meetings were offered, LEAG members opted to meet remotely, which may have limited opportunities for connection. LEAG meetings were held in a shared space, which may have been challenging for some participants due to the lack of privacy and potential discomfort in sharing personal experiences in a group setting. Therefore, it may have affected who we recruited into the group. The absence of a clear project timeline and research activity map may have negatively impacted ongoing engagement. Additionally, meetings were arranged pragmatically, which could have further affected consistent attendance. Furthermore, while LEAG members brought a range of experiences and perspectives, the group lacked diversity in terms of ethnicity and gender, a common challenge in PPIE work [33]. LEAG members were predominantly white British females, which does not represent the broader population. Evidence suggests that young people from ethnic minority groups may face a greater risk of TASA, highlighting the need for more inclusive group membership [34]. Despite partnering with a specialist organisation, our approach did not achieve the desired diversity. Promotional materials were not co‐developed with individuals from marginalised communities, which may have limited their reach. Future research should consider peer‐led outreach, collaboration with intersectional community organisations and co‐designed recruitment materials to improve representation.

In conclusion, this study demonstrates that meaningful co‐design of DHIs with people with lived experience is not only feasible but essential even when addressing sensitive and stigmatised topics like TASA. Our partnership with the LEAG yielded valuable insights that shaped both the content and functionality of the i‐Minds DHI, while highlighting important considerations for conducting PPIE work in digital health research. Our structured approach to co‐design enabled us to balance user needs with technical constraints while ensuring meaningful engagement. Despite challenges around diversity and sustained participation, our experience underscores the value of early lived experience involvement, the promotion of partnership with specialist organisations and lived experience charities, and flexible engagement approaches. The lessons learned and recommendations provided offer a practical framework for future researchers seeking to co‐design DHIs, particularly those addressing sensitive topics or working with vulnerable populations. Most importantly, our work demonstrates that when properly supported and meaningfully engaged, experts by experience can make invaluable contributions to digital health research while finding personal value and connection in the process. Young people affected by TASA often experience barriers to accessing traditional forms of mental health support. The co‐design of i‐Minds with LEAG members helps ensure that the intervention is both relevant and acceptable to its intended users. By centring the voices of young people with lived experience, this study reflects a broader societal commitment to inclusive, user‐driven service development. It also demonstrates the value of PPIE in addressing complex public health challenges through digital innovation.

## Author Contributions

**Sandra Bucci:** conceptualisation, funding acquisition, methodology, supervision, writing – original draft. **Alice Newton:** data curation, investigation, formal analysis, project administration, writing – original draft. **Pauline Whelan:** funding acquisition, methodology, writing – review and editing. **Kim Cartwright:** funding acquisition, project administration, data curation, investigation, supervision, writing – review and editing. **Prathiba Chitsabsean:** writing – review and editing, funding acquisition. **Simon Foster:** methodology, writing – review and editing. **Victoria Green:** funding acquisition, writing – review and editing. **Amanda Larkin:** writing – review and editing, project administration, data curation, investigation, supervision. **Rhiannon‐Faye McDonald:** data curation, investigation, writing – review and editing. **Ethel Quayle:** funding acquisition, writing – review and editing, supervision. **Matthias Schwannauer:** funding acquisition, supervision, writing – review and editing. **Victoria Selby:** data curation, writing – review and editing, investigation. **Filippo Varese:** funding acquisition, writing – review and editing, supervision.

## Ethics Statement

Ethical approval for the i‐Minds programme of work was obtained from the National Research Ethics Committee (REC) of West Scotland REC 4 (approval number 22/WS/0083) and overseen by an independent oversight committee.

## Consent

Written informed consent for research participants taking part in the study and for the publication of the study findings was obtained.

## Conflicts of Interest

Unrelated to this project, S.B. and P.W. are co‐founders of CareLoop Health Ltd, which develops and markets digital therapeutics for schizophrenia and a digital screening app for postnatal depression. S.B. also reports research funding from the National Institute for Health and Care Research (NIHR), Wellcome Trust and Medical Research Council (MRC). F.V. reports research funding from the NIHR. P.W. reports research funding from the NIHR and Wellcome Trust. R.F.M. and V.G. work for the Marie Collins Foundation, a third sector organisation that supports victims of technology‐assisted child sexual abuse.

## Supporting information

Supporting file ‐ Merged.

## Data Availability

Data is available upon reasonable request.
