# Supplementary material for: Reporting on a Partnership to Co‐Design a Digital Health Intervention With Young People Who Have Experienced Technology‐Assisted Sexual Abuse
Source: Health Expect. 2025 May 10;28(3):e70288. doi: 10.1111/hex.70288 (PMC12064989; doi:10.1111/hex.70288)
Supplement: Supplementary file 1 — Supporting file ‐ Merged. [file HEX-28-e70288-s001.docx]

**Supplementary File 1.** Information poster


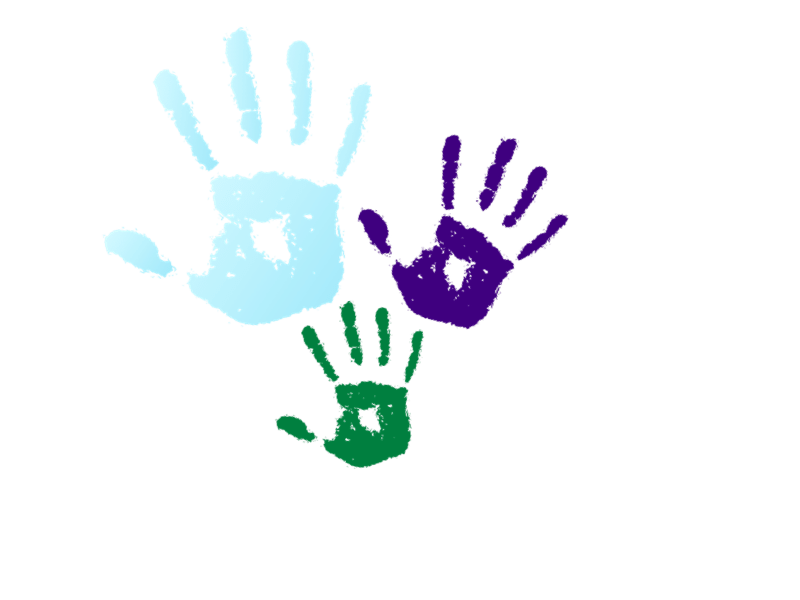
**Lived Experience Advisory Group for the i-Minds Project**

**We are conducting a research project called i-Minds. This project aims to help young people who have experienced unwanted online sexual experiences. As part of this research, we are setting up an advisory group. The group will be attended by young people who have had unwanted sexual experiences online to ensure the research is relevant and beneficial to young people. This information sheet will tell you a bit about the project and what being part of the advisory group will involve.**

**What is a lived experience advisory group?**

Lived experience can represent any type of experience; it means that you have lived through something and have gained knowledge and insight about that experience. Involving people with lived experience in research is very important. It ensures that research is carried out “by” and in “collaboration” with people, not done “to” or “about” people. This ensures research helps the people it is intended for in the best possible way and empowers those involved. It increases the likelihood of the right changes being made to the way services provide access to and deliver care to people, and in turn, helps ensure people’s needs are met and wellbeing improved. For the i-Minds project, we want to involve people who have had lived experience of unwanted online sexual experiences in how we carry out the research. Through the lived experience advisory group, we will gather a diverse range of perspectives about a range of issues, including how we develop the intervention we aim to test in this project, how to inform people about the research, and analysing and publicising the results of the research. We want to ensure that everything we do is in collaboration with individuals who have lived experience of unwanted online sexual experiences.

You can get further information about patient and public involvement in research at involve.org.uk

**What is the i-Minds project?**

i-Minds is a 27-month research project, funded by the National Institute for Health Research (NIHR). The aim of this research project is to work with young people, parents/caregivers and organisations and services that support young people to develop and test a digital resource to improve the well-being of young people who have had unwanted online sexual experiences, and help prevent them from being harmed again in the future.

Unwanted online sexual experiences involve a wide range of experiences and every person’s experience will be different. It could involve receiving unwanted sexual materials (e.g., photos, videos), inappropriate talking, and/or engaging in physical sexual activities in person following online communication.

Children and young people being exposed to unwanted online sexual experiences is increasing. Unfortunately, there are no specific resources to support children and young people who have had these experiences. In the i-Minds project, we want to create a resource that young people find easy to use and has a positive effect on mental health and well-being. We also want the resource to help young people stay safe online. The resource will be based on a type of talking therapy called “mentalisation therapy”, which has been shown to be helpful for young people. Mentalisation therapy is about how we understand other people’s thoughts, feelings, emotions, and behaviours. Sometimes it can be tricky to understand our own feelings and the feelings of those around us, especially when communicating online. Mentalisation therapy aims to make understanding other’s intentions easier. We hope this will have a positive impact on young people’s ability to better manage their emotions and risk of harm.

**What will I have to do if I do decide to be involved?**

As a member of our lived experience advisory group, you will be asked to attend group meetings every two months. Meetings will take place online (e.g. Zoom), but you will not be expected to use the camera function if you don’t want to. During the meeting, we will ask your opinion about how to conduct aspects of the research, how to best design the online resource, what information is important to include in the resource, and the language we should use to support people with unwanted sexual experiences online. Meetings will last approximately 2-3 hours and you will be reimbursed £40-£60 (£20 per hour) for your time. We will also reimburse you for any time spent reading materials (usually around 1 hour) we ask you to look through in preparation for and before meetings.

**Will I be asked about my lived experience?**

No, we will not ask about your personal experiences. You will not be asked to share anything you are not comfortable with.

**Am I eligible to take part?**

If you are age 18 and above and you have personal experience of unwanted online sexual experiences of any kind, you are eligible to take part in this advisory group.

**Are there any benefits to taking part?**

The lived experience advisory group will give you the opportunity to be heard and listened to. You will have the chance to shape a resource that can help young people who have had similar experiences to you. You will also be remunerated £20 per hour for your contribution; meetings will last 2-3 hours.

**What do we expect from group members?**

To make sure that every member gets the most out of attending meetings, it is important that everyone feels listened to. Members will have had a range of experiences and express different ideas, and all of these are valid and important. Everyone must be treated with respect.

**What do I do if I would like to join the group, or I want further information?**

You can contact Alice or Kim at the email addresses below, if you are interested in taking part. You can contact one of us and we will arrange a meeting where we can discuss what is involved in more detail and answer any questions you might have.

Thank you for taking the time to read this information sheet!

Contact details:

**Supplementary file 2.** Terms of Reference.

**i-Minds Young Persons’ Lived Experience Advisory Group**

**i-Minds**: A digital intervention to improve mental health and interpersonal resilience for young people who have experienced unwanted online sexual experiences.

**Brief project outline**

The aim of the i-Minds project is to work with young people, parents/caregivers and relevant organisations to first develop and then test a digital intervention to improve the well-being of young people who have experienced unwanted online sexual experiences. We want to create an intervention that: i) young people find easy to use; ii) has a positive effect on mental health and well-being and helps young people to stay safe on the Internet; and iii) could be used within NHS services that already support YP (e.g. Child and Adolescent Mental Health Services, CAMHS) and services that provide online support to young people. The project will consist of two work streams (Figure 1 below). In work stream 1, we will develop the intervention and in work stream 2 we will test delivering the intervention to young people who have experienced unwanted sexual experiences and find out what they think about it, for example, whether they found it useful and what they might change.

*Figure 1. Diagram to demonstrate work streams*

**Purpose and objective of the Lived Experience Advisory group**

The purpose of the i-Minds lived experience advisory group is to give the research team feedback, advice and guidance on all aspects of the research project to ensure that the research we conduct is appropriate and useful.

Members of the group may be asked to comment on things such as:

- How the intervention should be delivered, e.g. online or via an app.
- What language is most appropriate to use in relation to the experience of unwanted online sexual experiences.
- The structure of the intervention.
- How we word and phrase our research materials.

Members will never be asked for any details of their own lived experience, and at **NO POINT** will be expected to discuss their lived experience.

**Confidentiality - staff and members.**

It is important that the group (including any research staff that attend) upholds strict confidentiality at all times, not discussing anyone’s personal or private business outside of the group and/or within group where not appropriate.

Member details will not be shared with any other agencies or organisations. However, it is important to note that confidentiality can be broken where there is reason to believe that there is risk of significant harm to oneself or others.

We will take notes/minutes during the meeting. However, we will ensure that the notes we take during the meetings are anonymised and no details of any members are shared with anyone else. This includes ensuring that no group emails are sent in which members could see another member’s email address. We will *not* audio or video record the meetings.

**Minutes and notes**

We will be taking minutes from the meetings; this will include brief notes about what we have discussed. All of the minutes taken will be anonymised; they will not include any information about who has said what. They will also not include ANY personal discussion; they will purely stick to gathering the information you have given us regarding the project. For example, although members will never be asked for any details of their own lived experience, and at no point will be expected to discuss their lived experience, if members choose to discuss their lived experience, this will never be written in the notes / minutes.

The minutes will be shared with advisory group members via email after the group has taken place to that we are open and clear with you about the notes we have taken.

The anonymised minutes may be shared with other members of the i-Minds research team to give them the information they need to develop the intervention. The minutes from the meeting will not be shared with anyone outside of our research team. They will be stored on secure NHS Trust servers and password protected to make sure they are kept safe and secure.

**Membership**

The lived experience advisory group will have a maximum of 20 members, with representation from Manchester and Edinburgh (the two sites where the research is taking place) as well as from other regions in the UK. Membership of the lived experience advisory group will be open to those who are aged 18 and above who have been affected by unwanted online sexual experiences.

Membership of the lived experience advisory group is voluntary, but we ask that members commit to attending meetings and respond to emails/correspondence. When members are unable to attend meetings, contributions to the areas to be discussed can be shared via e-mail or post.

The i-Minds project reserves the right to review membership of the group and appoint members should a place become available.

**Process of membership**

The individual would make contact with a member of the i-minds project team. A member of the i-Minds team will then set up a time to discuss what taking part will involve in more detail.

**Meetings**

The i-Minds lived experience advisory group will aim to meet approximately every two months. Meetings will last no longer than 2-3 hours. The number of meetings may change depending on the needs of members and the project. Meetings will be held via Zoom. There may be an opportunity for face-to-face meetings in the future, depending on whether this can be facilitated or not.

Meetings, where possible, will be arranged with the members of the group to ensure that the time and date is suitable for as many members as possible. Members will also be given reasonable notice of the time and date of meetings.

**Remuneration**

Members will be remunerated £20 per hour for their time in line with the NIHR INVOLVE’s guidance. Please see link below if you would like further information.

<https://www.nihr.ac.uk/documents/reward-and-recognition-for-public-contributors-a-guide-to-the-payment-of-fees-and-expenses/12248>

This includes time spent on any activity related to participation in the group, for example, reading materials sent out in advance of meetings, preparing for meetings or any other activities carried out outside of meetings themselves that contribute to the group’s goals (e.g. attending events to publicise the findings of the project).

To receive payment, members need to complete a Bank Details Request Form to be set up on Greater Manchester Mental Health NHS Foundation Trust’s payroll / finance system. This form will be emailed to members in advance of or after the first meeting. After each meeting / activity members will need to complete a Volunteers and Non-Staff Claim Form. On this form, members need to provide details of the activities they were engaged in (e.g. reading in preparation of meeting, attending meeting), how long they spent on each activity, what the hourly rate is (i.e. £20 per hour), what the total they are owed is and sign and date the form. As this these forms are processed electronically, once members have completed one of these forms, with permission from the research team, for future activities / meetings the research team can fill in forms on behalf of members, provided members are happy for the research team to input their name and the date on the form. A member of the research team will submit forms to the NHS Trust’s finance / payroll department for processing and members will receive any remuneration directly into their bank account (i.e. via BACS).

The remuneration process will be explained to members at their first meeting and members can contact a member of the research team at any point should they have any questions about the process.

**Member responsibilities**

1. Members are expected to contribute within meetings where they feel comfortable to do so.
2. Members are expected to monitor their own well-being and let someone know if they feel uncomfortable with a group topic.
3. Members are expected to confirm attendance to meetings or send apologies 24 hours in advance of the meeting, where possible.
4. Members are expected to review any materials provided in a timely manner and attend meetings prepared.
5. Members are also expected to try and remain focused within meetings and keep discussions on track.

**Code of conduct (to be updated when discussed with the lived experience advisory group)**

1. At all times, group members are to treat each other with respect.
2. The group is to acknowledge that members will have range of different opinions and there are no “right” or “wrong” answers or ideas.
3. The group is to acknowledge that members will have a range of different skills, abilities, experiences and be supportive of each other if members need additional help with anything relevant to discussions / tasks being carried out by the group.
4. Members must work collaboratively with other group members and members of staff.
5. All persons present at meetings must try not to interrupt, if you need to speak, ask the chair of the meeting.

**What to do if you need support**

There might be times when difficult feelings come up either within or after meetings. We want to try to make sure that all members are supported with this. If you become upset within a meeting you are free to leave at any point. You are always free to contact a member of the team to discuss any problems you might be having with attending the group.

There will be a clinician available when the meetings are held, and 24 hours afterwards within working hours (9-5). You will be able to contact this person to discuss anything you feel you need to.

We would also encourage you to speak to any regular support workers or your GP if you are struggling.

**Terms of reference**

The terms of reference for the lived experience advisory group will be reviewed and updated continuously in collaboration with the group and the research team. This is to ensure that the group runs to its maximum effectiveness for both researchers and members.
